# Fever of unknown origin in B-cell depleted patients: Have you considered Neoehrlichiosis?

**DOI:** 10.1007/s10067-025-07394-z

**Published:** 2025-03-15

**Authors:** Lea-Kristin Nagler, Marc Schmalzing, Nora Isberner, Christoph Schoen, Michael Gernert

**Affiliations:** 1 Department of Medicine II, Rheumatology and Clinical Immunology, University Hospital of Würzburg, Oberdürrbacher Str. 6, 97080 Würzburg, Germany; 2 Department of Medicine II, Infectious Diseases, University Hospital of Würzburg, Würzburg, Germany; 3 Institute for Hygiene and Microbiology, Julius-Maximilians-Universität Würzburg, Würzburg, Germany

Rheumatologists frequently encounter cases of fever of unknown origin (FUO), whether in immunosuppressed patients with autoimmune diseases or in those referred for evaluation of persistent, unexplained fever. FUO is characterized by a prolonged febrile state with no identifiable cause after initial standard diagnostic approaches. First described over six decades ago, FUO continues to be a significant challenge, requiring comprehensive and often invasive diagnostic investigations to uncover the underlying cause [1]. The differential diagnosis is broad, encompassing infectious, neoplastic, autoimmune, and miscellaneous etiologies [2]. Advances of the recent years, such as targeted therapies, and emerging pathogens, have expanded the scope of potential culprits.

This case series includes six patients (three female, three male; aged 49–80 years) with underlying rheumatoid arthritis, granulomatosis with polyangiitis (GPA), and multiple sclerosis (MS) that presented with FUO (Table 1). Five had received rituximab for rheumatologic disease; one was treated with ocrelizumab for MS. Symptoms preceding diagnosis ranged from 2 to 14 months, and included fever, fatigue, night sweats, and weight loss. Initial investigations, including laboratory tests, imaging, endoscopy, and bone marrow analysis, ruled out infections and malignancies. Four patients were hospitalized. Notable findings were splenomegaly in three patients, thrombocytopenia in two, and elevated CRP levels in all cases. Despite comprehensive diagnostics, the cause of FUO remained unidentified. Finally, eubacterial polymerase chain reaction (PCR) testing of venous blood samples revealed evidence of *Candidatus Neoehrlichia mikurensis*, confirming the diagnosis of Neoehrlichiosis. Targeted antibiotic therapy with doxycycline was initiated, leading to significant clinical improvement. Fever resolved, and symptoms gradually subsided. Follow-up imaging demonstrated a reduction in splenomegaly and normalization of blood count.

**Table 1:** Characteristics of patients with Neoehrlichiosis

Case	A	B	C	D	E	F
Sex	f	f	m	f	m	f
Age, *years*	49	58	64	63	80	57
Underlying disease	Rheumatoid arthritis, T-CUS	GPA	GPA	Rheumatoid arthritis	GPA	MS
Type of immunsuppression	Rituximab	Rituximab	Rituximab	Rituximab, MTX	Rituximab	Ocrelizumab
B-lymphocytes, */µl* ^a^	0	0	126	0	338	0
IgG, *mg/dl* ^a^	839	448	708	345	990	*unknown*
Duration of symptoms, *months*	9	3	8	9	2	14
Symptoms						
Fever	yes	yes	yes	yes	yes	yes
Night sweats	yes	yes	yes	yes		yes
Weight loss		yes	yes	yes	yes	
Fatique	yes	yes	yes	yes		yes
Other		Cough	Thrombosis	Cough		
Clinical findings						
Splenomegaly	yes		yes			yes
Blood work-up	Thrombocytopenia			Anemia, thrombocytopenia		
CRP (mg/dl), max	10.11	9.54	4.32	1.49	12.0	2.85
Evaluation of FUO						
Suspected diagnosis	T-LGL	Respiratory tract infection	Occult malignancy	Occult malignancy	Infection	Infection, occult malignancy
Non-conclusive work-up^b^	*Laboratory testing:* Serological testing for HIV, hepatitis viruses, HSV, VZV, EBV, CMV *Imaging:* Ultrasound of the abdomen, CT of the chest/ abdomen, TTE, TEE *Other:* Bone marrow aspirate 3 hospitalizations	*Laboratory testing:* Serological testing for hepatitis viruses, HSV, VZV, EBV, CMV, HHV6 *Imaging:* Ultrasound of the abdomen, CT of the chest, TEE *Endoscopy:* Bronchoscopy 1 hospitalization	*Laboratory testing:* No additional testing *Imaging:* Ultrasound of the abdomen, CT of the chest *Endoscopy:* Gastroscopy, colonoscopy, cystoscopy	*Laboratory testing:* Serological testing for HIV, HSV, VZV, EBV, CMV and candida, aspergillus, pneumocystis *Imaging:* X-ray of the chest, CT of the abdomen, TTE, TEE *Endoscopy:* Gastroscopy *Other:* Gynecologic and dermatological screening	*Laboratory testing:* No additional testing *Imaging:* Ultrasound of the abdomen, CT of the chest and sinuses 1 hospitalization	*Laboratory testing:* Serological testing for zoonotic illnesses, PCR testing for EBV, CMV, testing for rheumatologic disorders, vaginal smear *Imaging:* Ultrasound of the abdomen; CT of the chest/ abdomen, TTE; TEE; FDG PET-CT *Other:* Bone marrow aspirate, consultation of dentistry, ENT, gynecology, urology 2 hospitalizations
Unsuccessful empiric treatment	Intensification of immunosuppressive therapy (GCs, Baricitinib, cyclophosphamide)	2 courses of antibiotic therapy (Amoxicillin/ clavulanic acid, Moxifloxacin)				5 courses of antibiotic therapy (Trimethoprim/ sulfamethoxazole, Clindamycin, 2× Amoxicillin/ clavulanic acid, Moxifloxacin)

*f* Female; *m* Male; *T-CUS* T cell clones of uncertain significance; *T-LGL* T cell large granular lymphocyte leukemia; *GPA* Granulomatosis with polyangiitis; *MS* Multiple sclerosis; *MTX* Methotrexate; *IgG* Immunglobulin G; *CRP* C-reactive protein; *max* Maximum; *TTE* Transthoracic echocardiography; *TEE* Transesophageal echocardiography; *FUO* Fever of unknown origin; *GCs* Glucocorticoids; *ENT* Ear, nose, and throat; *PCR* Polymerase chain reaction; *FDG PET-CT* Fluorodeoxyglucose positron emission tomography–computed tomography

^a^ Last assessment before diagnosis of Neoehrlichiosis

^b^ Basic laboratory testing included: complete blood count, complete metabolic panel, CRP, urinalysis, blood cultures, SARS-CoV2 testing

Neoehrlichiosis is a rare disease caused by the tick-borne obligate intracellular bacterium *Candidatus Neoehrlichia mikurensis* predominantly affecting immunocompromised individuals. The bacterium from the order *Rickettsiales* is transmitted through *Ixodes* ticks, commonly found in Central and Northern Europe and Asia [3]. Although our patients resided in high-risk areas (Bavaria, Germany), none reported active tick bites at diagnosis. The infection’s latency period remains unclear. Symptoms are nonspecific, including fever, fatigue, night sweats, and weight loss. Severe cases may present with thromboembolic events or vasculitis. Patients on B-cell-depleting therapy are particularly vulnerable, alongside those receiving other immunosuppressive treatments (e.g., glucocorticoids, TNF-α inhibitors) or with hematologic disorders. Immunocompetent individuals typically experience milder disease [3–5]. The association between B-cell depletion and susceptibility to Neoehrlichiosis is underexplored. Diagnosis relies on PCR testing, as the bacterium is currently uncultivable and commercial serology is unavailable. Treatment typically involves prolonged courses of doxycycline since most standard antibiotics are ineffective [4].

Conventional diagnostic methods, such as blood cultures and serology, often fail to detect rare pathogens in immunosuppressed patients. Eubacterial 16S rRNA PCR, which amplifies bacterial DNA directly from clinical samples, overcomes these limitations and offers a broad detection range [6]. However, the accessibility of this method is limited, as it is not widely available. The 16S rRNA PCR test was designed to detect more than 100 rDNA copies per milliliter, reducing the risk of false-positive results [7]. To ensure accuracy, sequences exceeding 700 bases are compared with the GenBank database, and phylogenetic analysis is performed using the BIBI web server [8]. Test specificity for bacteria and fungi is generally ≥ 98%, following CLSI guidelines [9]. However, due to the lack of a validated reference test for *Candidatus Neoehrlichia mikurensis*, precise clinical sensitivity and specificity remain undetermined. In our cases, no other significant pathogens were detected using 16S rRNA PCR. The presented cases underscore the vulnerability of B-cell-depleted patients to rare infections like Neoehrlichiosis, emphasizing the need for heightened clinical awareness, particularly in high-risk areas for tick-borne diseases. In these endemic regions, clinicians should consider *Candidatus Neoehrlichia mikurensis* in FUO, particularly when conventional methods fail. Combining molecular diagnostics with traditional methods enhances accuracy, enabling timely treatment while minimizing unnecessary investigations, hospital stays, and inappropriate antibiotic use. Recognizing the evolving infectious risks associated with advanced immunosuppressive therapies is crucial for optimizing care in this high-risk population.
